# Extracellular proteins as potential biomarkers in Sepsis-related cerebral injury

**DOI:** 10.3389/fimmu.2023.1128476

**Published:** 2023-10-13

**Authors:** Jinlan Dong, Shuang Wang, Zhonghua Hu, Li Gong

**Affiliations:** Department of Anesthesiology, The Third Xiangya Hospital of Central South University, Changsha, Hunan, China

**Keywords:** septic encephalopathy, bioinformatics analysis, extracellular protein, protein-protein interactions, biomarkers

## Abstract

**Background:**

Sepsis can cause brain damage known as septic encephalopathy (SAE), which is linked to higher mortality and poorer outcomes. Objective clinical markers for SAE diagnosis and prognosis are lacking. This study aimed to identify biomarkers of SAE by investigating genes and extracellular proteins involved in sepsis-induced brain injury.

**Methods:**

Extracellular protein differentially expressed genes (EP-DEGs) from sepsis patients’ brain tissue (GSE135838) were obtained from Gene Expression Omnibus (GEO) and evaluated by protein annotation database. The function and pathways of EP-DEGs were examined using GO and KEGG. Protein-protein interaction (PPI) networks were built and crucial EP-DEGs were screened using STRING, Cytoscape, MCODE, and Cytohubba. The diagnostic and prognostic accuracy of key EP-DEGs was assessed in 31 sepsis patients’ blood samples and a rat cecal ligation and puncture (CLP)-induced sepsis model. Cognitive and spatial memory impairment was evaluated 7-11 days post-CLP using behavioral tests. Blood and cerebrospinal fluid from 26 rats (SHAM n=14, CLP n=12) were collected 6 days after CLP to analyze key EP-DEGs.

**Results:**

Thirty-one EP-DEGs from DEGs were examined. Bone marrow leukocytes, neutrophil movement, leukocyte migration, and reactions to molecules with bacterial origin were all enhanced in EP-DEGs. In comparison to the sham-operated group, sepsis rats had higher levels of MMP8 and S100A8 proteins in their venous blood (both *p*<0.05) and cerebrospinal fluid (*p*=0.0506, *p*<0.0001, respectively). Four important extracellular proteins, MMP8, CSF3, IL-6, and S100A8, were identified in clinical peripheral blood samples. MMP8 and S100A8 levels in the peripheral blood of sepsis patients were higher in SAE than in non-SAE. In comparison to MMP8, S100A8 had a higher area under the curve (AUC: 0.962, *p*<0.05) and a higher sensitivity and specificity (80% and 100%, respectively) than MMP8 (AUC: 0.790, *p*<0.05). High levels of S100A8 strongly correlated with 28-day mortality and the Glasgow Coma Scale (GCS) scores.

**Conclusion:**

The extracellular proteins MMP8, CSF3, IL-6, and S100A8 may be crucial in the pathophysiology of SAE. S100A8 and MMP8 are possible biomarkers for SAE’s onset and progression. This research may help to clarify the pathogenesis of SAE and improve the diagnosis and prognosis of the disease.

## 1 Introduction

Sepsis is an infection-related syndrome that affects various organ systems in the body (1). With more than 30 million individuals diagnosed yearly, sepsis is one of the leading etiologies of death in critically ill patients globally (2). In 2017, 11 million septicemic-related deaths were reported, accounting for 19.7% of all deaths globally, while there were approximately 49 million cases of septicemic events globally (3). Even if the sepsis patient survives, the high expense of sepsis treatment imposes a strain on the countries and individuals’ finances. Of all the organs and systems attacked by sepsis, the brain acts as a susceptible target and bears the brunt known as sepsis-associated encephalopathy (SAE). SAE occurs in roughly 70% of sepsis patients and is associated with a higher risk of death and poorer long-term outcomes (4, 5). SAE is an intertwined condition characterized as a state of neurological disturbances induced by systemic reactions to infection, central nervous system (CNS) elimination infection, and other factors (6). Further, a broad spectrum of neurological symptoms, including long-term cognitive impairment and psychological effects, is also present in survivors (e.g., anxiety and depression) (7). SAE predicts a poor prognosis, and mortality in individuals with SAE increases with severity, up to 70% (5). It has drawn progressively increasing attention in recent years.

While the exact pathophysiology of SAE remains to be elucidated, several hypothesized mechanisms have been proposed and examined (8). Neuroinflammation (9), oxidative stress, impaired cerebral perfusion and blood microcirculation (10), vascular endothelial dysfunction (11), and other variables, according to mounting evidence, have been linked to the main pathophysiological mechanisms of SAE. These factors can be triggered and regulated by various extracellular signaling molecules. In the extracellular environment of the central nervous system, these molecules, primarily secreted proteins, are dispersed in body fluids and tissues and have immunomodulatory features that facilitate immune cell interactions and neuroinflammatory cascade (12). Furthermore, these humoral indicators provide a novel approach for diagnosing and tracking SAE, particularly for patients who cannot undergo a clinical examination (13). These could be more sensitive to the actual degree of brain damage in SAE compared to comprehensive neuropsychiatric assessment and time-consuming brain MRI (14). The development of SAE, brain dysfunction and death have all been associated with serum markers of brain injury, including the protein S100A8, neuron-specific enolase, and the N-terminal pro-peptide of NT-proCNP (15). S100β has been found to promote microglia and astrocyte activation in sepsis, which triggers neuronal death (16). Numerous studies have also shown that several pro-inflammatory cytokines, which serve as biomarkers of inflammation and oxidative stress, such as interleukins (IL-1 and IL-4) and tumor necrosis factor (TNF) (17), rise sharply after infection and enter the central nervous system, promoting brain edema and vascular injury in response to the attack of pathogens and their toxins. These cytokines are also signs of sepsis-induced brain injury. But rare investigated the sources of these biomarkers present in body fluids and tissues.

A novel strategy to investigate the pathophysiology of SAE, identify clinical biomarkers, and find therapeutic targets is a bioinformatic analysis of transcriptional patterns in microarrays. Numerous studies on the sepsis transcriptome have found notable alterations in the gene expression profiles in the peripheral blood of sepsis patients, indicating that differentially expressed mRNAs and the proteins they encode may be engaged in the mechanisms of sepsis development (18–20). And some studies utilized transcriptomic data to perform clustering analysis and identified different subtypes of sepsis that are associated with clinical severity and mortality (21, 22). Due to the dearth of brain tissue samples from sepsis patients, little has been known about the expression profiles of sepsis patients’ brain tissue. However, the environment in which the lesions are present may most accurately reflect the disease’s clinical process. Consequently, it is crucial to understand the processes and indicators of sepsis by analyzing the expression profiles of brain tissue in sepsis patients. We hypothesized that the rat model will provide additional insights into the molecular mechanisms underlying sepsis at the level of CSF in CNS. This work sought to discover vital extracellular proteins that might contribute to the pathogenesis of SAE, look for biomarkers that might aid in the clinical diagnosis and prognosis of sepsis-related brain injury, and investigate potential new therapeutic targets.

## 2 Materials and methods

### 2.1 Acquisition of microarray data and processing for the differentially expressed genes and genes differentially expressed for extracellular proteins

The transcriptome characteristics of the postmortem parietal cortex were compared between 12 sepsis deaths and 12 non-septic critically sick individuals in the GEO database GSE135838. The GEO database GSE135838 is available on the National Center for Biotechnology Information (NCBI) website at https://www.ncbi.nlm.nih.gov/geo/query/acc.cgi.acc=GSE135838. The entire RNA data from the chosen samples were downloaded for subsequent analysis, but information on each sample’s age, etiology, medication treatment, or prognosis was unavailable. The “Deseq2” R package was then applied to the raw data to identify the genes differentially expressed in human brains between septic patients and non-septic critically ill patients. In each sample, genes that met the following requirements remained (1): |log2 (fold change) | > 1 and (2) *p*< 0.05. With the help of the “ggplot2” R package, a volcanic map was made to illustrate the results. Additionally, we used the “pheatmap” R package to conduct a bi-directional hierarchical clustering analysis of the collected DEGs, using TBtools to visualize the result as a heat map. Then, the Human Protein Atlas (HPA) protein annotation database and the Uniprot database were used to download the extracellular protein gene list GO:0005576 and the extracellular protein gene list, respectively. To identify the most commonly occurring EP-DEGs, these two lists were intersected with the DEGs from the GSE135838 dataset using a Venn diagram. The significantly highly expressed EP-DEGs were then displayed on a heat map.

### 2.2 Assessment of cerebral mRNA and plasma protein levels of EP-DEGs in healthy

The HPA Human brain dataset was used to evaluate the mRNA expression levels of the 14 top EP-DEGs, which include the cerebral cortex and the hippocampal formation in the healthy brain in 12 different regions. Expression stereograms were created using the nTPM values from the database. The HPA dataset’s protein concentration values for the 14 highly expressed EP-DEGs in normal plasma, which has been reported in multiple literature sources, were also used to construct the scatter figure.

### 2.3 Gene set enrichment analysis, gene ontology enrichment and Kyoto Encyclopedia of Genes and Genomes pathway analysis

DAVID Bioinformatics Resources v6.8 was used to do the Gene Ontology (GO) mapping and Kyoto Encyclopedia of Genes and Genomes (KEGG) pathways analysis to annotate the potential functions of EP-DEGs. The projected biological process (BP), cell composition (CC), and molecular function (MF) of each EP-DEG were evaluated, and all the EP-DEGs were posted to the DAVID website (version 6.8). The enriched GO terms were displayed using the R programming language’s GOplot package, which was also used to examine the intersection of the genes implicated in the GO terms. Additionally, the pathways in which the EP-DEGs participated were predicted and mapped using the KEGG database in conjunction with DAVID.

### 2.4 Protein-protein interaction network analysis and identification of hub genes

The Search Tool for Retrieval of 31 Interacting EP-DEG (STRING; http://STRING-db.org;GeneMANIA; http://genemania.org/) online database was used to create protein network interaction maps. These two-genome PPI networks were predicted using the multi-protein online tool in the STRING database, and they were displayed using Cytoscape 3.6.1. The most critical modules were then filtered using the Complex Detection (MCODE) plugin, a Cytoscape App, which recognizes clusters in the produced network based on topology and detects strongly connected regions. Then, the MCC and Radiality algorithm in Cytohubba, an APP in Cytoscape, was utilized to screen out the hub genes (23). To map the protein interaction network and further investigate which biological processes the gene cluster is engaged in, the genes in the gene cluster with the highest score were screened and imported into STRING.

### 2.5 Verification of potential biomarker expression in both cerebrospinal fluid and blood of sepsis rat

As previously reported (24), sepsis was induced using cecal ligation and puncture (CLP) in male Sprague-Dawley rats weighing 280 ± 30 g under sterile conditions with the use of anesthesia. After midline laparotomy, the cecum was exposed, ligated below the ileocecal valve, and punctured twice with a sterile 18-gauge needle. The abdominal cavity was closed with sutures following CLP, and normal saline (5 ml/250 g body weight) was injected subcutaneously. The control group underwent a sham operation with laparotomy and suturing only. The experimental protocol was approved by the Animal Use and Ethics Committee of Xiangya Third Hospital, Central South University (Approval NO. XMSB-2023-0018).

The induction of sepsis in rats leads to varying degrees of cognitive impairment and subsequent behavioral changes. It is critical to observe the behavior of rats to validate biomarkers and evaluate the successful establishment of the SAE model (25, 26). The modeled rats were randomly divided into two groups, one for behavioral testing (8 in the sham group and 11 in the CLP group) and the other for sample collection testing (14 in the sham group and 12 in the CLP group). Behavioral tests, including novel object recognition (NOR) and the Barnes maze test, were performed to determine the cognitive functions of rats after modeling. Referring to previous reference (27), rats were habituated to a NOR test arena without objects for one day, and then placed in a testing arena containing familiar and novel objects. These objects were randomly used as either familiar or novel, without repetition. Exploration behavior was recorded during each session. By calculating the novel object recognition index, the experiment assesses the memory capacity of mice in recognizing new objects. This index is obtained by dividing the time spent exploring the new object by the total time spent exploring both the new and familiar objects. The assessment of spatial learning ability in rats is performed using the Barnes maze, according to earlier research (28). Rats are positioned on a circular platform that contains 20 equally spaced holes, with one of them leading to a dark chamber referred to as the target box. To locate the target box, rats are stimulated with powerful light (200 W) while on the platform. The rats’ performance is evaluated in the reference memory phase on the first day following the training sessions, which involves recording the number of errors they make during each attempt using a video tracking system.

After 6 days of CLP modeling, cerebrospinal fluid and blood samples were collected from the rats under deep anesthesia using a stereotactic instrument, with the rats placed in a 45° prone position with their heads facing upward. The skin and muscle were incised after neck dissection, and a needle was inserted through the dura mater to collect 100-200 μl of CSF from the medullary canal (approximately 0.2 mm in depth). Subsequently, the chest was opened, and 4 ml of blood was collected from the left ventricle. The collected CSF was centrifuged at 2000g for 20 minutes at 4°C, and the collected blood was centrifuged at 3000g for 10 minutes. The obtained supernatants were collected and stored at -80°C for further analysis. ELISA was performed to validate the expression levels of EP-DEG in the control and septic blood samples. The levels of MMP8 and S100A8 were determined using commercial ELISA kits (MMP8: Cat. #ER1166, Wuhan Fine Biotech Co., Ltd., China; S100A8: Cat. #ER0592, Wuhan Fine Biotech Co., Ltd., China) according to the manufacturer’s instructions.

### 2.6 Study population and clinical data collection verification of potential biomarker expression in sepsis patients

Patients 18 years of age or older who met the clinical criteria for severe sepsis (1) within the previous 24 hours at any time during their stay in the ICU were enrolled in the study after being screened for eligibility criteria. Non-infected critically ill patients within 24 hours of admission were enrolled as the control group. All participants provided written informed consent, and our study was approved by the Ethical Review Committee of the third Xiangya Hospital, Changsha, China (Approval number: 22139) and registered at the Chinese Clinical Trial Registry (ChiCTR2300069425). The following were the exclusion criteria: primary central nervous system disease, such as cerebral hemorrhage; pre-existing psychiatric or neurological disorders; use of immunosuppressive medications, such as antibiotics and hormones before admission; recent history of blood transfusion; clear evidence of encephalitis/meningitis; altered consciousness and mental status due to other causes of acute encephalopathy; renal replacement therapy, such as hemofiltration and hemodialysis; cancer. Clinical data were collected on all recruited cases, including vital signs, blood gas analysis, blood routine, liver and kidney function, infection site, mechanical ventilation duration, and more. The patients’ SOFA, APACHE-II, and GCS scores were evaluated. 28-day mortality, ICU length of stay, and hospitalization time were tracked. All individuals underwent a neuropsychiatric evaluation for brain damage. A thorough history from the patient or family was acquired to look for early clinical indicators of brain dysfunction, such as disorientation, agitation, or a loss of consciousness. All SAE diagnoses come from the records of ICU patients’ medical histories by ICU physicians. In the ICU, SAE refers to the clinical manifestations of concomitant extra-infection and neurological dysfunction in sepsis patients, which can present as impairment of consciousness ranging from delirium to coma. Doctors need to perform neurological system examination and consciousness state assessment for sepsis patients, and exclude other acute cerebrovascular diseases, metabolic encephalopathy, intoxication, alcohol or drug withdrawal, pulmonary brain disease, non-convulsive epileptic seizures, and failure of peripheral organs such as heart, liver and kidney that could cause similar symptoms, to make an accurate diagnosis of SAE. Non-SAE patients are those who have sepsis but have not developed encephalopathy.

**Figure 1:**
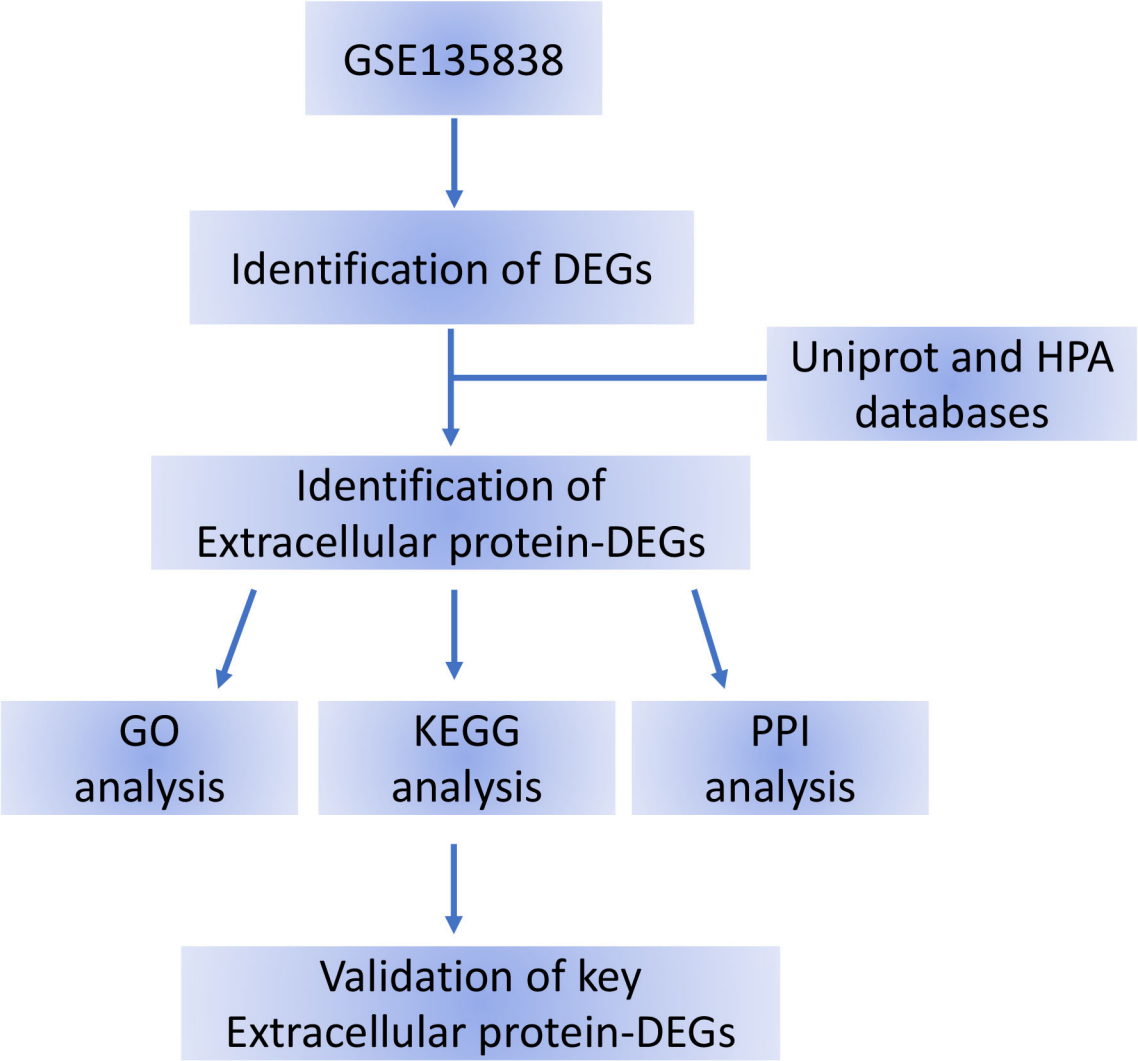
Flow chart of the bioinformatics analysis. DEG, differentially expressed gene; HPA, Human Protein Atlas; DEGs, differentially expressed genes.

Peripheral venous blood was drawn three milliliters within 24 hours after admission. Following the manufacturer’s recommendations, an ELISA was carried out to verify the expression levels of EP-DEG in the control and sepsis blood samples. The levels of IL6, MMP8, CSF3, and S100A8 were determined by the following commercial ELISA kits, according to manufacturer’s recommendations: MMP8 (Cat.#CSB-E04680h, Cusabio Biotech Co. Ltd, Wuhan, China), S100A8 (Cat. #CSB-E11833h, Cusabio Biotech Co. Ltd, Wuhan, China), IL-6 (Cat. #CSB-E04638h, Cusabio Biotech Co. Ltd, Wuhan, China), and CSF3 (Cat. #EK0360, Boster Biotech Co. Ltd, USA). The flow chart for this study is shown in **Figure 1**.

The pivot gene’s diagnostic value was estimated using ROC analysis, which was carried out in R using the pROC package (29). The hub gene is regarded to have strong specificity and sensitivity in differentiating between non-SAE and SAE when the AUC value is more than 0.7.

### 2.7 Statistical methods

The Biobase package, limma package, and ClusterPro “ler package of R were used to evaluate mRNA expression from GEO database. SPSS 26.0 and GraphPad V9.1.1 software were utilized to do statistical analysis on clinical data. The mean differences between the two groups were compared using a two-tailed independent samples t-test. Receiver operating characteristic (ROC) curves were used to obtain the performance characteristics of diagnostic SAEs. The area values under the curve were used in the ROC curve study. Pearson correlation analysis was used to assess the relationship between the levels of different EP-DEGs and clinical indicators as well as prognostic factors, including mechanical ventilation duration, 28-day mortality, ICU length of stay, and hospitalization time. All *p* values were two-tailed, and statistical significance was set at *p*< 0.05.

## 3 Results

### 3.1 Sample information processing and identification of DEGs

**Figure 2:**
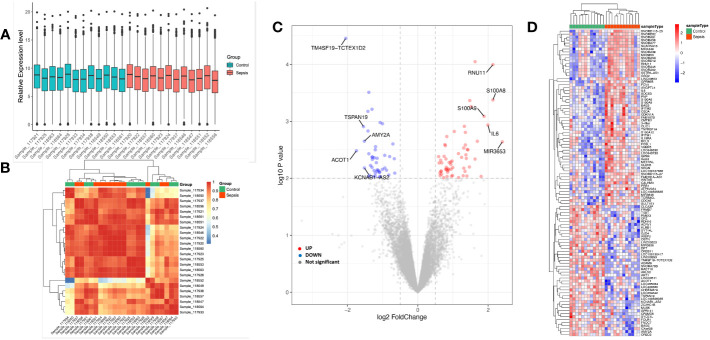
Sample data processing and identification of DEGs in the dataset between the sepsis group and the control group. Gene probe expression levels across samples are shown in **(A)** a boxplot. The median, higher quartile, and lower quartile did not significantly differ from one another. **(B)** Heatmap showing sample-to-sample correlation. The intra-group correlation in the sepsis group was stronger than it was in the control group. **(C)** Volcano map of all DEGs in the control and sepsis groups that were examined using the limma R tool. On the map, the top 10 genes that were up- and down-regulated and had the lowest p-value are displayed. **(D)** Heatmap displaying all DEGs in both the sepsis group and the control group.

A between-sample differential gene expression analysis was carried out to assess the variations in gene expression between the sepsis group and the critical ill control group. In the GSE135838 dataset, the median, upper and lower quartiles, maximum and minimum values of the 24 sample genes were virtually identical (**Figure 2A**). In the sepsis group, correlation analysis revealed a more significant intra-group association (**Figure 2B**). According to the sample information and data matrix analysis of GSE135838, 165 differentially expressed genes were extracted from the brain tissues. TM4SF19-TCTEX1D2, CPZ, ADAM6, ZC2HC1B, and ART1 were down-regulated genes, while SNORD115-27, RNU11, S100A8, SNORA45, LOC649330, S100A9, and IL6 were the top seven up-regulated genes with the least *p*-values (**Figure 2C**). The heat map of DEGs revealed that, in contrast to the control group, DEGs were consistently up or downregulated in the sepsis group (**Figure 2D**).

### 3.2 Screening of EP-DEGs

**Figure 3:**
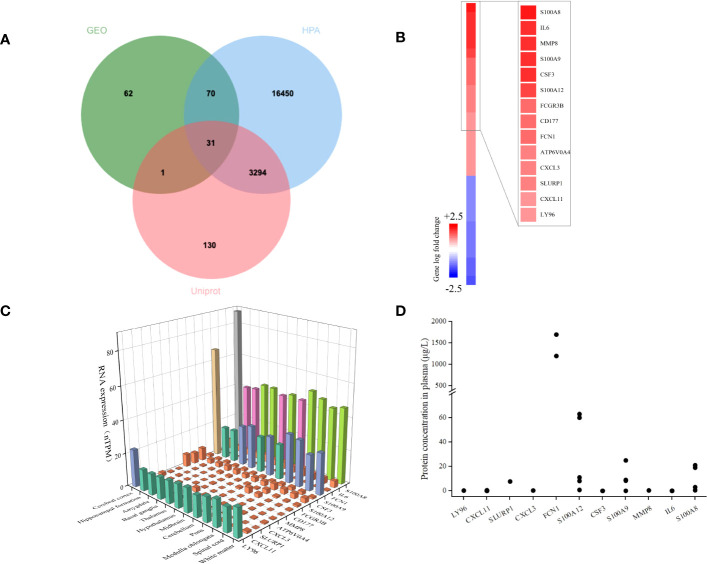
Identification of DEGs encoding extracellular proteins. **(A)** The genes encoding extracellular proteins annotated in the Uniprot and HPA database were intersected with DEGs, and 31 EP-DEGs were screened; 19 were found to be up-regulated and 12 to be down-regulated. **(B)** A heatmap of EP-DEGs in the sepsis group and control group. The top 14 elevated genes with the lowest p-value were highlighted. **(C)** A three-dimensional histogram of mRNA expression of the top 14 upregulated genes in various regions of the normal brain. **(D)** Plasma concentrations of 12 extracellular proteins in a healthy population.

We used extracellular protein genes annotated in existing public libraries to filter EP-DEGs from DEGs. Extracellular protein genes that were identified in the HPA and Uniprot databases were intersected with DEGs, and 31 EP-DEGs were screened; 19 were found to be up-regulated and 12 to be down-regulated (**Figure 3A**). Among the top 14 up-regulated extracellular protein-encoding genes in the sepsis and control groups that had the highest logFC values were S100A8, IL6, MMP8, S100A9, CSF3, S100A12, FCGR3B, CD177, FCN1, ATP6V0A4, CXCL3, SLURP1, CXCL11, and LY96 (**Figure 3B**). To further analyze these genes, their mRNA and protein levels in healthy individuals were summarized. A three-dimensional histogram was used to show how the top 14 up-regulated genes’ mRNA expression varied in different parts of the normal brain (**Figure 3C**). MMP8, CXCL3, SLURP1, CXCL11, and FCN1 had relatively low amounts of mRNA in normal brain tissue according to HPA database. Still these levels were markedly increased in the brain tissues of sepsis patients. Except for CD177 and ATP6V0A4, which were not reported, all 12 extracellular proteins among the top 14 up-regulated genes were expressed at various levels in the plasma of healthy populations. FCN1 expression was the highest among all, reaching 1.7 mg/L. The scatter plot shows how these 12 plasma proteins are distributed in terms of concentration across the literature (**Figure 3D**). Among the extracellular molecules CSF3, IL6, MMP8, and S100A8, no significant correlations (*p*<0.05) as shown in **Figure S1**.

### 3.3 Functional and pathway enrichment analysis of EP-DEGs

**Figure 4:**
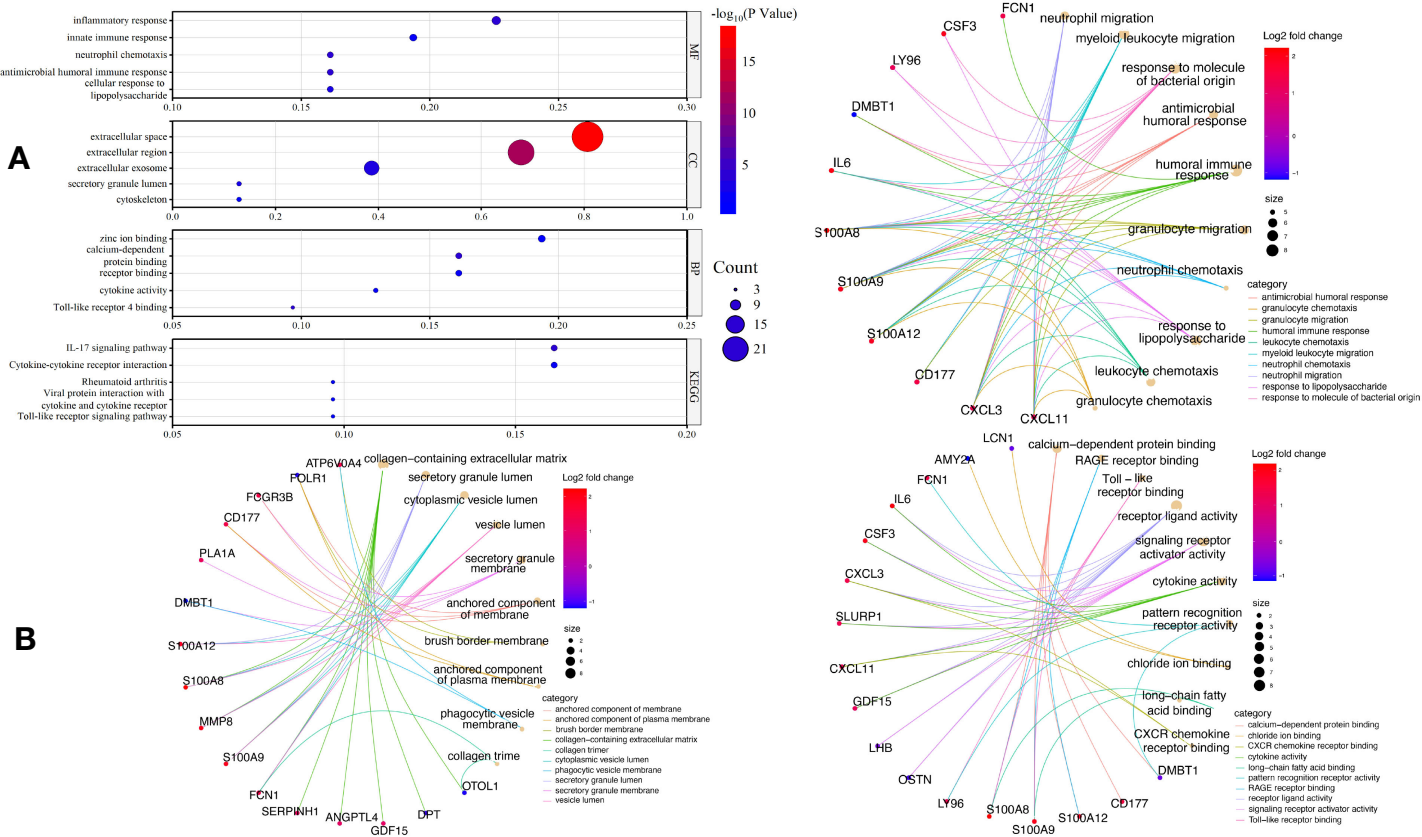
GO and KEGG enrichment of EP-DEGs. **(A)** The top five enriched GO terms for BPs, CCs, MFs, as well as five enriched KEGG processes are shown in the dotplots. **(B)** Circle graph in the GO enrichment of the EP-DEGs. The top five GO categories for BPs, CCs, and MFs, respectively, for which the EP-DEGs are enriched, are shown in the circle graph. The colored points serve as a representation of the GO categories, and the size of a point indicates the number of genes it accumulates. The color of the line that a point deliver corresponds to the category of the point in the legend.

To comprehend the function of the screened 31 EP-DEGs discovered in the brain tissue dataset, functional and pathway enrichment analyses were performed using DAVID. As seen in **Figure 4A**, these genes are primarily enriched in biological processes such as zinc ion binding, calcium-dependent protein binding, and cytokine activity. Inflammatory response, innate immune response, and neutrophil chemotaxis are primarily enriched in these genes. All the genes are enriched in extracellular space. All genes are enriched in the KEGG pathway of the IL-17 signaling pathway, cytokine-cytokine receptor interaction, and other signaling pathway. The top 5 processes with the minimum *p*-value of BPs, CCs, and MFs are displayed using the cnetplot function in the ClusterProfiler package. GDF15, LY96, CSF3, IL6, CD177, CXCL3, S100A8, S100A9, S100A12, and FCN1 are enriched in at least two BPs, CCs, and MFs (**Figure 4B**).

The significantly enriched hallmark terms revealed by GSEA included defense response to other organisms, regulation of response to stress, and others (**Figure S2**).

### 3.4 Gene cluster identification and protein-protein interaction network analysis

**Figure 5:**
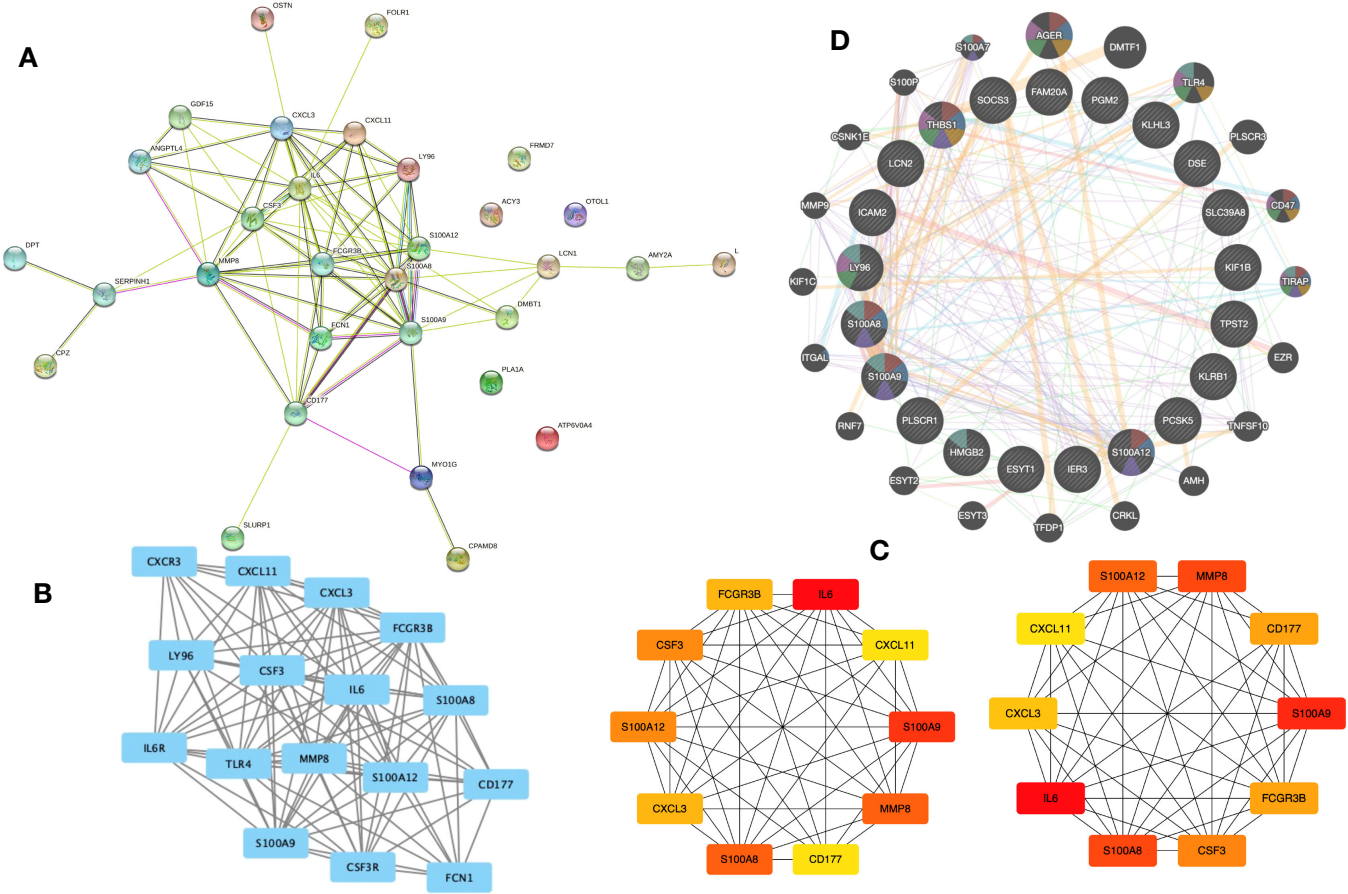
Building a PPI network of EP-DEGs and identifying hub genes. **(A)** The 26 nodes and 86 edges of the PPI network of EP-DEGs are created using the STRING database. **(B)** The node gene cluster created by Cytoscape’s MCODE plug-in with the highest score comprises 16 genes. **(C)** The top ten hub genes constructed by the Cytohubba. **(D)** According to GeneMANIA analysis, the roles of 31 EP-DEGs were mainly in myeloid leukocyte, leukocyte migration, neutrophil migration, and response to molecules of bacterial origin.

We built a PPI network using the STRING database to investigate how the 31 EP-DEGs interact with one another. Utilizing the cytoscape application, the PPI network was seen (**Figure 5A**). The PPI network consists of 86 edges and 26 nodes using calculations of molecular pathways and processes linked to the 31 core DEGs. We developed working modules using the MCODE plug-in for Cytoscape. The findings showed a module of 16 genes (**Figure 5B**). Using ten topological techniques from the CytoHubba plug-in in Cytoscape, the top 10 hub genes were screened. The four genes MMP8, CSF3, IL-6, and S100A8 were included in each of the two methods (**Figure 5C**). Results of GeneMANIA also revealed that the functions of 31 EP-DEGs were primarily related to myeloid leukocyte, leukocyte migration, neutrophil migration, and response to molecules of bacterial origin (**Figure 5D**).

### 3.5 MMP8 and S100A8 diagnostic and prognostic efficacy

**Figure 6:**
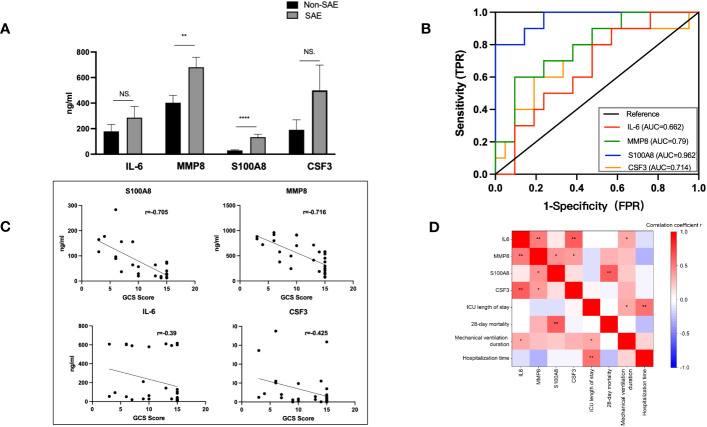
Levels of IL-6, CSF3, MMP8, and S100A8 and ROC curves of S100A8 and MMP8 in the peripheral blood of SAE patients. **(A)** When compared to the non-SAE (n=10) group, the SAE patients (n=21) had higher serum levels of MMP8 and S100A8 (both *p*<0.0). **(B)** High diagnostic accuracy was shown for the identification of SAE by both S100A8 (AUC = 0.962, 95% CI = 0.9001-1.000) and MMP8 (AUC = 0.7905, 95% CI = 0.6245-0.9564), with S100A8 having the highest accuracy. **(C, D)** Correlation analysis of the four extracellular molecules with clinical indicators and prognostic markers. S100A8, MMP8, CSF3, and IL-6 protein levels in the peripheral blood of sepsis patients were significantly linked with GCS scores (all *p*<0.05). S100A8 levels and 28-day mortality were strongly associated (r=0.634, *p*<0.01), and IL-6 levels and duration of mechanical breathing were positively correlated (r=0.360, *p*=0.046). *p <0.05, **p <0.01, ****p <0.0001; ns, no significance.

31 sepsis patients, including 10 SAE patients and 21 non-SAE patients, were enrolled to confirm the main extracellular proteins found. Serum samples from the patients were taken to measure the levels of MMP8, CSF3, IL-6, and S100A8. Additionally, 31 sepsis patients’ clinicopathological characteristics, such as the source of the sepsis, the medical history, their comorbidities, and other variables, were compared between patients with and without SAE (**Table 1**). The SAE group exhibited notably higher scores for SOFA compared to the non-SAE group (*p*<0.05). Between the SAE and non-SAE group, there were no appreciable differences in the source of sepsis as well as the medical history. Septic shock (*p*=0.049), acute renal injury (*p*=0.0067), and acute lung injury (*p*=0.0129) were all more prevalent in SAE patients. The 28-day mortality rate was higher in the SAE group, with a *p*-value of 0.0501 that was considered marginally significant. The concentrations of the proteins S100A8, MMP8, CSF3, and IL-6, were measured in peripheral blood samples from 31 sepsis patients to validate the critical extracellular proteins identified in the study. The SAE patients had higher levels of IL-6, MMP8, and S100A8 when compared to the non-SAE (n=10) group (all *p*<0.05) (**Figure 6A**). The predictive usefulness of MMP8 and S100A8 in the diagnosis and prognosis of SAE was investigated using ROC curves. Both S100A8 (AUC = 0.962, 95% CI = 0.9001-1.000) and MMP8(AUC = 0.791, 95% CI = 0.625-0.956) demonstrated high diagnostic accuracy for the diagnosis of SAE, with S100A8 having the highest AUC (**Figure 6B**). S100A8 had a cut-off value of 82.29 pg/ml and corresponding sensitivity and specificity of 80% and 100%, respectively. S100A8, MMP8, CSF3 and IL-6 protein levels in the peripheral blood of sepsis patients were revealed to be substantially associated with GCS scores following a correlation investigation of the four extracellular molecules with clinical indications (all *p*<0.05) (**Figure 6C**). S100A8 levels were significantly correlated with 28-day mortality (r=0.634, *p*<0.01) and IL-6 levels were positively correlated with mechanical ventilation time (r=0.360, *p*=0.046), according to a correlation analysis of the four extracellular molecules and clinical prognostic markers (**Figure 6D**).

**Table 1:** Clinicopathological characteristics of 31 sepsis patients.

Patient demographics and laboratory information	non-SAE(n=21)	SAE(n=10)	*p*
Average age (year)	65.38	61.4	0.4685
Sex (female)	7 (14)	6 (4)	0.1596
Mechanial ventilation, n	5 (16)	5 (5)	0.1448
Vasopressor administration, n	5 (16)	6 (4)	0.049^*^
APACHE-II score, mean SOFA score, mean	9.611 4.905	14.000 8.500	0.0786 0.0277^*^
GCS score, mean	14	6	<0.0001^****^
Source of sepsis			0.8226
Pneumonia	3	2	
Urinary tract	5	3	
Intra-abdominal	12	4	
Other sources	1	1	
History			0.7053
Diabetes, n	4	2	
Hypertension, n	7	3	
Chronic lung disease, n	2	1	
Coronic artery disease, n	1	2	
Comorbidities			
Septic shock, n	5 (16)	6 (4)	0.049^*^
Septic cardiomyopathy, n	2 (19)	3 (7)	0.1473
Acute renal injury, n	8 (13)	9 (1)	0.0067^**^
Acute hepatic injury, n	7 (14)	5 (5)	0.3732
Acute lung injury, n	9 (12)	9 (1)	0.0129^*^
Outcomes			
ICU mortality, n	0 (21)	0 (10)	
28-day mortality, n	1 (19)	3 (7)	0.0501
ICU length of stay (days)	4.311	4.35	0.9874
Mechanical ventilation duration (hours)	13.39	39.6	0.1443
Hospitalization time (days)	12.86	16.3	0.463

^*^p<0.05, ^**^p<0.01, ****p<0.0001.

**Figure 7:**
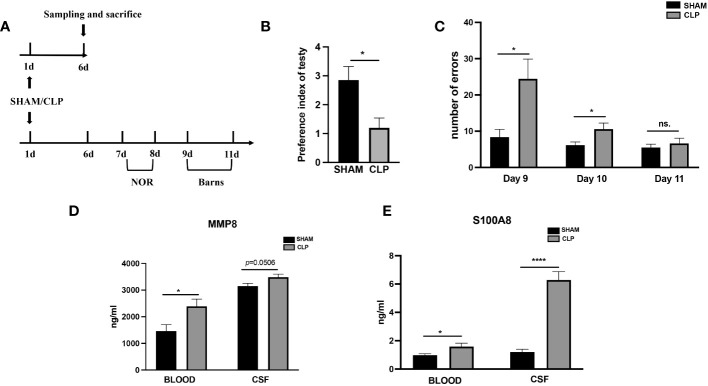
Levels of MMP8, and S100A8 in the peripheral blood and CSF of sepsis rats. **(A)**Timeline of experiment procedures. **(B)** The recognition index of novel items in the CLP model rats (n =11) was lower than that of the Sham group rats (*p* < 0.05 vs Sham, n = 8). **(C)** In the Barnes maze experiment, the CLP model rats (n =11) made significantly more mistakes before finding the target hole on postoperative day 9 and 10 compared to the Sham group rats (*p* < 0.01, *p* < 0.05 *vs* Sham, n = 8). **(D)** The CLP rats (n=14) exhibited significantly elevated serum levels of MMP8 (*p*<0.05) and CSF levels (*p*=0.0506) in comparison to the SHAM group (n=12). **(E)** When compared to the SHAM (n=14) group, the CLP rats (n=12) had higher serum levels of S100A8(*p*<0.01) and CSF levels (all *p*<0.0001). CLP, cecal ligation and puncture; NS., no significance; NOR, novel object recognition. *p <0.05; ****p <0.0001.

Following a successful modeling procedure, a total of 45 rats, comprising the SHAM group (n=22) and the CLP group (n=23), were utilized for behavioral experiments and for obtaining blood and CSF samples (**Figure 7A**). The CLP model rats (n=11) exhibited a significantly lower novel object recognition index compared to the SHAM group rats (n=8) seven to eight days after modeling (*p <*0.05 *vs*. Sham) (**Figure 7B**). In the Barnes maze experiment, CLP model rats (n=11) made significantly more errors before locating the target hole on the 9^th^ and 10^th^ day after surgery than SHAM group rats (n=8) (*p <*0.01, *p <*0.05 *vs.* Sham) (**Figure 7C**). Modeling induced brain injury and septic encephalopathy in the rats. The concentrations of S100A8 and MMP8 proteins were measured in the rat blood and CSF samples obtained six days after modeling to confirm the key extracellular proteins identified in the study. In comparison to the SHAM group (n=14), CLP rats (n=12) had significantly increased serum MMP8 levels (*p <*0.05), with CSF levels also increasing (*p* = 0.0506) (**Figure 7D**). Similarly, compared to the SHAM group (n=14), the serum S100A8 levels in CLP rats (n=12) were higher (*p <*0.01), with significant increases in CSF levels (*p* <0.0001) (**Figure 7E**).

## 4 Discussion

Sepsis is a syndrome of organ dysfunction involving multiple organ systems throughout the body resulting from a dysregulated response to infection and can be caused by infection at any site (1, 30). Cerebral dysfunction in sepsis patients is frequently a sign of a high mortality rate (31). Finding a trustworthy approach to identify SAE is crucial from a clinical standpoint since it can help with prognosis prediction, organ support and protection, early brain injury detection, and disability reduction. The medical history, neurological physical examination, scoring, biochemical testing, neurological imaging, transcorporeal Doppler ultrasonography, electroencephalography, and pathology can all be used to identify the current diagnosis of SAE (32). However, it is challenging to make an early diagnosis of cerebral dysfunction due to the invasiveness, lack of specificity, and costly equipment requirements of these procedures. Early detection and definitive diagnosis of sepsis have significant implications for reducing healthcare expenditures and mortality from sepsis (33).

The most likely indicator of septic brain injury is the presence of extracellular secretory proteins in the brain or CSF (6, 34, 35). CSF is more difficult to inventory than blood, though. Because extracellular secreted proteins like cytokines and chemokines can pass the blood-brain barrier, they may represent neuroimmune events in the central nervous system (36–39). S100A8/9, IL-1, IL-4, and tumor necrosis factor have all been identified in previous research as markers of sepsis-related brain damage in plasma (40–43). This sheds light on the possibility that some genes and the proteins they encode are preferentially expressed in SEA. Sepsis brain tissue microarray gene expression profiles (GSE135838) were obtained and analyzed using the GEO database to search for DEGs across brain tissue samples from critically sick controls and septic patients. Genes that were EP-DEGs were then chosen from the DEGs. Utilizing the GO and KEGG databases, the biological pathways, and functions of EP-DEGs were enhanced. As a result, we discovered several significant genes through the construction of PPI networks and configurations, opening up new possibilities for sepsis-related pathophysiology and prognosis. Clinical specimens were used to validate the relationship between potential biomarkers and sepsis-related brain damage.

Previous studies have reported that sepsis-induced brain injury is closely associated with inflammatory response, neutrophil chemotaxis, and cytokine pathways (44). Consistent with these findings, our KEGG and GO enrichment analysis of the 31 screened EP-DEGs revealed their significant enrichment in these biological processes, which are closely related to immune response. Through the construction and analysis of PPI networks and modules, this study further identified several genes that have the potential to be significant for the diagnosis, prognosis, and therapeutic targeting of SAE.

PPI identified top hub genes (MMP8, CSF3, IL-6, and S100A8) that are associated with myeloid lineage cells, leukocyte migration, neutrophil migration, and response to bacterial molecules. They may mediate the occurrence and development of SAE through these immune cells and immune activities. MMP8 and S100A8 expression have been linked to conditions including tumors, inflammation, and so on (45, 46), but these genes may be novel targets for SAE. Interestingly, we found that MMP8 and S100A8 may be potential biological biomarkers for the diagnosis and prognosis of SAE, based on a study of 31 carefully selected infected patients and 45 rat models. Through behavioral tests on model rats, sepsis induction in rats resulted in SAE in our animal experiments. This study is the first to compare levels of CNS and peripheral EPs in the SAE model. And higher levels of both S100A8 and MMP8 were shown in CSF compared to peripheral blood, with S100A8 exhibiting a more pronounced change in CSF compared to the SHAM group.

Our findings also indicated that the sepsis group rats exhibited poorer behavioral performance than the SHAM group and had higher levels of S100A8 and MMP8 in both blood and cerebrospinal fluid samples, suggesting that elevated levels of secreted proteins S100A8 and MMP8 may serve as indicators of impaired brain function. In addition, higher concentrations of MMP8 and S100A8 in the sera of SAE patients, compared to non-SAE, exhibited a favorable connection with GCS scores. This leads us to believe that blood levels of MMP8 and S100A8 may be valuable biomarkers of sepsis-related brain damage. Plasma S100A8 levels had the strongest correlation with the GCS scores of all these markers. This agrees with the findings of earlier research on S100A8 and septic encephalopathy (47). S100A8 is consistently highly expressed in the septic brain, according to several studies. It plays a role in regulating neuroinflammation by promoting granulocyte infiltration, activating microglia in response to reactive oxygen species, and producing cytokines (48). In this investigation, we also evaluated the significance of the four extracellular molecules for the prognosis of SAE. Even though just four instances in our collection passed away within 28 days, there was a significant correlation between S100A8 levels and 28-day mortality. This suggests that S100A8 is also a predictor of the prognosis for SAE patients. The blood’s granulocytes and monocyte-macrophages can be produced, differentiated, and perform certain functions under the regulation of the cytokine CSF3 (49). The fact that this CSF3 increases granulocytes and monocytes-macrophages and is closely linked to sepsis is undeniable. However, according to our findings, CSF3 did not have diagnostic efficacy as good as S100A8 and MMP8 in SAE. It was reported that peripheral treatment of CSF3 showed neuroprotective characteristics in models of TBI, Parkinson’s disease, and carbon monoxide poisoning (50–53). Our study cannot yet verify that CSF3 possesses neuroprotective properties. Future studies will need to verify it. MMP-8, a collagenase made by neutrophils, catalyzes both type I collagen and non-collagenous proteolysis, such as creating of pro-inflammatory chemokines. Previous clinical studies have shown that systemic proteolysis, a new fundamental pathological mechanism in septic shock, is associated with patient mortality. One of the primary factors that contribute to systemic protein breakdown is the heightened activity of MMPs (54). Furthermore, septic shock patients had elevated levels of blood MMP-8 mRNA and protein (54, 55).

MMP8 is a crucial mediator of neuroinflammation and controls microglia activation. Previous research indicates that MMP-8 is a fundamental cause of blood-brain barrier (BBB) disruption and neuronal damage in human bacterial meningitis (56). And high serum levels of MMP-8 are associated with catastrophic outcomes following cerebral trauma. In healthy individuals, plasma MMP8 protein levels are modest; nevertheless, in sepsis, particularly in those who have had a combined brain injury, plasma MMP8 levels are considerably raised and are correlated with the degree of distant brain function (57). This suggests that MMP8 may function as a biological marker of brain impairment brought on by sepsis. Upon measuring the levels of S100A8 and MMP8 simultaneously in the cerebrospinal fluid and peripheral venous blood of septic rats, we found their concentrations to be significantly higher in the cerebrospinal fluid compared to the blood. The possible explanation for the elevated expression of these proteins in peripheral is that in SAE, the BBB may become compromised, allowing proteins that are usually restricted to the CNS to leak into the peripheral circulation.

This research has several restrictions. The results could be skewed by the limited sample size because there is currently less publically available data on gene regulation from human brain tissue than from human peripheral blood. As it is unknown whether these septic patients suffered from SAE and brain injury in GSE135838, it is possible that the DEGs screened out may not adequately indicate brain damage. Further research on gene expression in brain tissue from SAE patients is required. Second, we employed two protein annotation databases to filter extracellular proteins; nevertheless, most protein localization information for both databases came from published studies. This evidence is insufficient, and extracellular proteins might be absent. Additionally, monitoring markers like EEG monitoring that more accurately indicate brain function are not present in the clinical cases we gathered. In addition, we did not receive CSF samples that would have clearly shown a decline in brain function due to the patients’ and families’ rejection. Shock, APACHE II, GCS, hospital mortality, PCT, and CRP all point to septic individuals having a more severe state than other patients. We cannot rule out the possibility that the severity of sepsis just serves as a risk factor for SAE and a confounder of the association between SAE and each plasma protein level. Lastly, this study could not reveal the origin of the peripheral proteins. More study is needed to achieve these goals.

This study combined clinical and bioinformatic evidence to identify potential SAE biomarkers. 31 EP-DEGs were screened, and their involvement in biological processes and pathways was anticipated by examining the gene expression profiles of human brain tissue in sepsis. Four core plasma secretory proteins, MMP8, CSF3, IL-6, and S100A8, were identified. Clinical blood samples were utilized to verify the concentrations of these four proteins. The diagnostic and prognostic accuracy of MMP8 and S100A8 was evaluated using clinical peripheral blood samples, CSF, and behavioral experiments in a rat model of sepsis. S100A8 and MMP8 are possible biomarkers for SAE’s onset and progression. This research may help to clarify the pathogenesis of SAE and improve the diagnosis of the disease.

## Data availability statement

The original contributions presented in the study are included in the article/**Supplementary Material**, further inquiries can be directed to the corresponding author/s.

## Ethics statement

The studies involving human participants were reviewed and approved by the Ethics Committee of Xiangya Hospital of Central South University (permit NO. 202112257). Written informed consent for participation was not required for this study in accordance with the national legislation and the institutional requirements.

## Author contributions

ZH and LG conceived the idea, searched relevant information, and performed all bioinformatics analysis. JD and SW harvested the clinical samples and record and arrange the clinical data. LG,JD, and SW performed the ELISA examination, drafted, and finalized the article. All authors contributed to the article and approved the submitted version. All authors contributed to the article and are recognized for the submitted version.

## Glossary

**Table:**

APACHE II	Acute Physiology and Chronic Health Evaluation II
AUC	Area Under Curve
BBB	blood-brain barrier
BP	biological process
CC	cell composition
CLP	cecal ligation and puncture
CSF	cerebrospinal fluid
DEGs	differentially expressed genes
EP- DEGs	Extracellular protein differentially expressed genes
GCS	Glasgow Coma Scale
GEO	Gene Expression Omnibus
GEPIA	Gene expression profiling interactive analysis
GO	Gene Ontology
GSEA	Gene Set Enrichment Analysis
HPA	Human Protein Atlas
KEGG	Kyoto Encyclopedia of Genes and Genomes
MF	molecular function
MMSE	Mini-Mental State Examination
NOR	novel object recognition
PPI	Protein-protein interaction
TBI	traumatic brain injury
TPM	Transcripts Per Million
SAE	septic encephalopathy
SOFA	Sequential Organ Failure Assessment
