# Supplementary material for: Extracellular proteins as potential biomarkers in Sepsis-related cerebral injury
Source: Front Immunol. 2023 Oct 13;14:1128476. doi: 10.3389/fimmu.2023.1128476 (PMC10611492; doi:10.3389/fimmu.2023.1128476)
Supplement: Supplementary file 1 [file DataSheet_1.pdf]

Supplementary Materials

1.Figures: 3

2. R programming language code

Figure S1. Correlation heatmap of correlations among CSF3, IL6, MMP8, and S100A8.

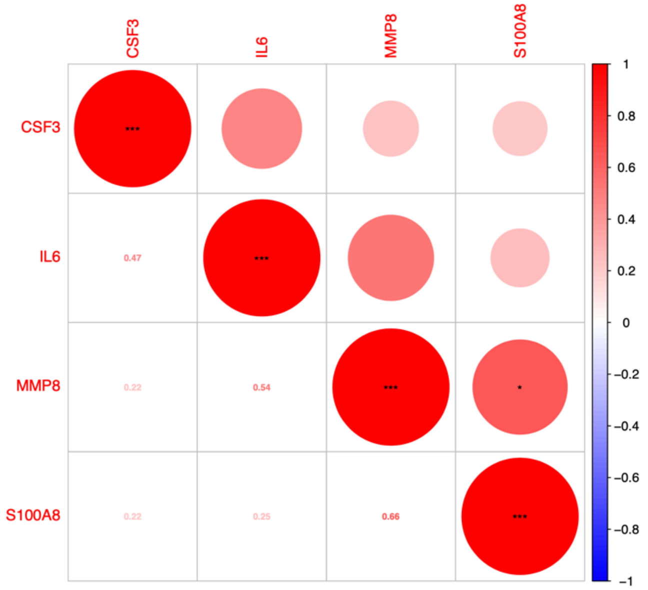

Figure S2. The visualization of some important enrichment hallmark terms by Gene Set Enrichment Analysis (GSEA).

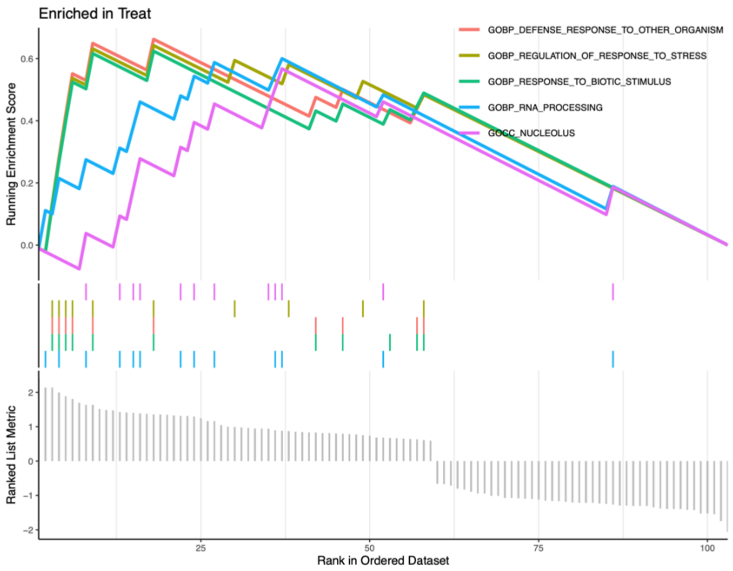

Figure S3. Hub genes were identified by LASSO regression analysis. (A) LASSO coefficient graph of ANGPTL4, ATP6V0A4, CPZ, CSF3, DMBT1, DPT, FOLR1, FRMD7, MYO1G, OTOL1, PLA1A and SLURP1. (B) The error rate curve.

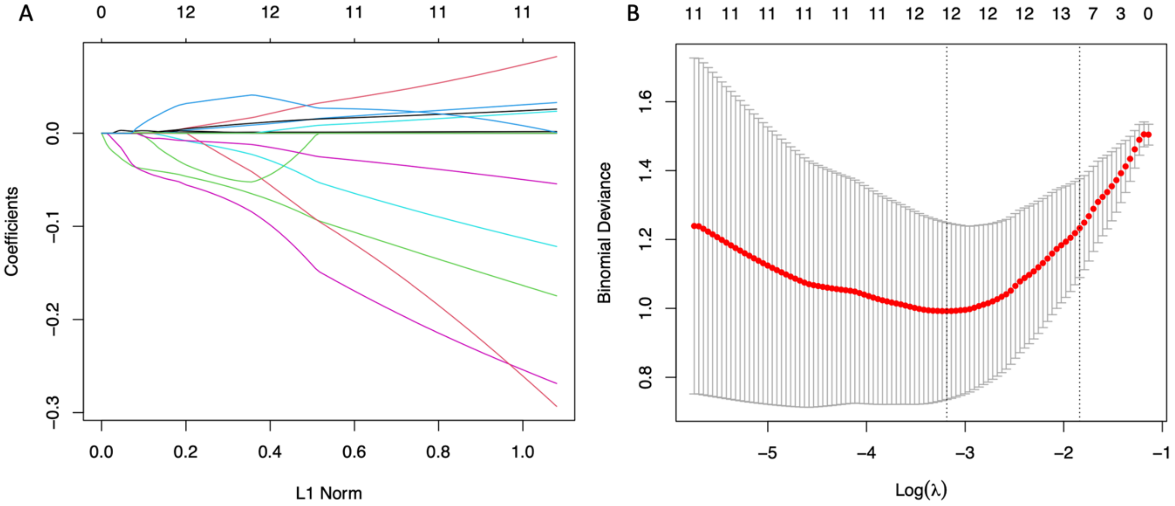

R programming language code

1.Correlation

library(corrplot)　#引用包

inputFile="diffGeneExp.txt"　#差异基因的表达文件

setwd("D:\\桌面\\新建文件夹\\cor")　#设置工作目录

#读取输入文件

```
data=read.table(inputFile, header=T, sep="\t", check.names=F, row.names=1)

#删除正常样品
group=gsub("(.*)\\_(.*)", "\\2", colnames(data))
rt=data[,group=="treat",drop=F]

#得到相关性矩阵
rt=t(rt)
M=cor(rt)
#进行相关性检验, 得到显著性 pvalue
res1=cor.mtest(rt, conf.level = .95)

#绘制相关性图形
pdf(file="corpot.pdf", width=7, height=7)
corrplot(M,
        type = "upper",       #图形展示在右上方
        method = "circle",    #图形以圆圈的形式展示
        col=colorRampPalette(c('blue', 'white', 'red'),alpha = TRUE)(100), tl.pos="lt",   #颜色的设置
        p.mat=res1$p, insig="label_sig", sig.level = c(.001, .01, .05), pch.cex = 0.85)   #加上显著性标记
corrplot(M, type="lower", add=TRUE, method="number",col=colorRampPalette(c('blue', 'white', 'red'),
alpha = TRUE)(100), tl.pos = "n", cl.pos="n", diag=FALSE, number.cex = 0.6)
dev.off()
```

2.GSEA

```
#引用包
library(limma)
library(org.Hs.eg.db)
library(clusterProfiler)
library(enrichplot)

inputFile="diff.txt"        #输入文件
gmtFile="c5.go.v7.4.symbols.gmt"      #基因集文件
setwd("D:\\桌面\\新建文件夹\\GSEA")      #设置工作目录

#读取文件,并对输入文件进行整理
rt=read.table(inputFile, header=T, sep="\t", check.names=F)
logFC=as.vector(rt[,2])
names(logFC)=as.vector(rt[,1])
logFC=sort(logFC, decreasing=T)
```

```
#读入基因集文件
gmt=read.gmt(gmtFile)

#富集分析
kk=GSEA(logFC, TERM2GENE=gmt, pvalueCutoff = 1)
kkTab=as.data.frame(kk)
kkTab=kkTab[kkTab$p.adjust<0.05,]
write.table(kkTab,file="GSEA.result.txt",sep="\t",quote=F,row.names = F)

#输出实验组富集的图形
termNum=5      #展示通路的数目
kkUp=kkTab[kkTab$NES>0,]
if(nrow(kkUp)>=termNum){
	showTerm=row.names(kkUp)[1:termNum]
	gseaplot=gseaplot2(kk, showTerm, base_size=8, title="Enriched in Treat")
	pdf(file="GSEA.treat.pdf", width=7, height=5.5)
	print(gseaplot)
	dev.off()
}

#输出正常组富集的图形
termNum=5      #展示通路的数目
kkDown=kkTab[kkTab$NES<0,]
if(nrow(kkDown)>=termNum){
	showTerm=row.names(kkDown)[1:termNum]
	gseaplot=gseaplot2(kk, showTerm, base_size=8, title="Enriched in Control")
	pdf(file="GSEA.con.pdf", width=7, height=5.5)
	print(gseaplot)
	dev.off()
}
```

3.LASSO analysis

```
 set.seed(12345)
library(glmnet)     #引用包
inputFile="diffGeneExp.txt"       #输入文件
setwd("C:\\biowolf\\geo\\lasso")      #设置工作目录

#读取输入文件
rt=read.table(inputFile, header=T, sep="\t", check.names=F, row.names=1)
rt=t(rt)
```

```
#构建模型
x=as.matrix(rt)
y=gsub("(.*)\\_(.*)", "\\2", row.names(rt))
fit=glmnet(x, y, family = "binomial", alpha=1)
cvfit=cv.glmnet(x, y, family="binomial", alpha=1,type.measure='deviance',nfolds = 10)
#绘制 Lasso 回归的图形
pdf(file="lasso.pdf", width=6, height=5.5)
plot(fit)
dev.off()
#绘制交叉验证的图形
pdf(file="cvfit.pdf",width=6,height=5.5)
plot(cvfit)
dev.off()

#输出筛选的特征基因
coef=coef(fit, s=cvfit$lambda.min)
index=which(coef != 0)
lassoGene=row.names(coef)[index]
lassoGene=lassoGene[-1]
write.table(lassoGene, file="LASSO.gene.txt", sep="\t", quote=F, row.names=F, col.names=F)
```
